# A Western-style diet exacerbates male infertility in *M1ap* knockout mice

**DOI:** 10.1093/humrep/deag130

**Published:** 2026-08-31

**Authors:** Brendan J Houston, Joseph Nguyen, Nadja Rotte, Frank Tüttelmann, Corinna Friedrich, D Jo Merriner, Anne E O’Connor, Moira K O’Bryan, Jessica E M Dunleavy

**Affiliations:** School of BioSciences and Bio21 Molecular Science and Biotechnology Institute, The University of Melbourne, Parkville, VIC, Australia; School of BioSciences and Bio21 Molecular Science and Biotechnology Institute, The University of Melbourne, Parkville, VIC, Australia; Institute of Reproductive Genetics, Centre of Medical Genetics, University of Münster, Münster, Germany; Institute of Reproductive Genetics, Centre of Medical Genetics, University of Münster, Münster, Germany; Institute of Reproductive Genetics, Centre of Medical Genetics, University of Münster, Münster, Germany; School of BioSciences and Bio21 Molecular Science and Biotechnology Institute, The University of Melbourne, Parkville, VIC, Australia; School of BioSciences and Bio21 Molecular Science and Biotechnology Institute, The University of Melbourne, Parkville, VIC, Australia; School of BioSciences and Bio21 Molecular Science and Biotechnology Institute, The University of Melbourne, Parkville, VIC, Australia; School of BioSciences and Bio21 Molecular Science and Biotechnology Institute, The University of Melbourne, Parkville, VIC, Australia

**Keywords:** M1AP, male infertility, genetic variants, Western diet, sperm quality

## Abstract

**STUDY QUESTION:**

What are the individual and combined effects of a common genetic cause of human male infertility (loss of *M1AP* function) and a negative lifestyle factor (a Western diet) on male fertility, spermatogenesis, and sperm quality in mice?

**SUMMARY ANSWER:**

The loss of M1AP and a Western diet separately led to subfertility (a form infertility) in male mice and in combination subfertility was worsened.

**WHAT IS KNOWN ALREADY:**

Genetics and lifestyle factors individually play important roles in male infertility aetiology. How lifestyle factors influence common genetic causes of infertility are unknown.

**STUDY DESIGN, SIZE, DURATION:**

We generated knockout *M1ap* mice (*M1ap^−/−-^*) and investigated the effects of genotype (wild type or knockout) and diet (control or Western diet) or genotype and age (young or aged) on male fertility and sperm parameters.

**PARTICIPANTS/MATERIALS, SETTINGS, METHODS:**

C57BL6/J mice: wild type (*M1ap^+/+^*) and *M1ap* knockout (*M1ap^−/−^*) at 10–12 (young) weeks or 26–28 weeks old (aged), on a control of Western (high-fat and high-sugar) diet and age. We evaluated male fertility, testis and epididymis histology, and sperm quality (motility, morphology, and count).

**MAIN RESULTS AND THE ROLE OF CHANCE:**

Compared to the individual gene deletion or diet treatments, the combined *M1ap^−/−^* and Western diet group had more abnormal germ cells, sloughed germ cells and resulted in fewer pups per litter. In accordance, sperm motility and morphology in the combination group were more significantly compromised compared to individual experimental groups.

**LARGE SCALE DATA:**

N/A.

**LIMITATIONS, REASONS FOR CAUTION:**

While the mutant mouse mimics the genetic variants seen in many infertile men, this is a mouse model study, meaning findings should be validated in a human cohort where possible.

**WIDER IMPLICATIONS OF THE FINDINGS:**

Taken together, our data highlight that genetic and lifestyle factors can interact to exacerbate male infertility. This study sets a precedent for an improved consideration of how genetic, lifestyle and environmental factors contribute to infertility aetiology. These data inform a clinical foundation for lifestyle interventions for men presenting at infertility clinics prior to medically assisted reproduction.

**FUNDING:**

This project was supported by a Society for Reproductive Biology Career Development Award to B.J.H., National Health and Medical Research Council project grant (APP1120356) awarded to M.K.O.B., an Australia–Germany Joint Research Co-operation Scheme (UA-DAAD) grant (57511796) awarded to M.K.O.B., J.E.M.D., and F.T., and the Deutsche Forschungsgemeinschaft (DFG, German Research Foundation) within the Clinical Research Unit ‘Male Germ Cells’ (CRU326, project number 329621271) awarded to F.T. and C.F.

**DISCLOSURES:**

N/A.

## Introduction

Male infertility is a common, highly heterogeneous disease that impacts at least 7% of men worldwide (Krausz and Riera-Escamilla, 2018). As predicted by the many thousands of genes expressed in the testis, including many that are enriched in expression compared to other tissues, male infertility has a strong genetic component (Schultz *et al.*, 2003; Djureinovic *et al.*, 2014; Fagerberg *et al.*, 2014; Uhlen *et al.*, 2015; Wyrwoll *et al.*, 2020; Houston *et al.*, 2021a, 2024b; Nagirnaja *et al.*, 2022; Oud *et al.*, 2022). Compelling data indicate that lifestyle and environmental exposures also play an important role in shaping the environment within which sperm are formed, and thus male fertility (Durairajanayagam, 2018; Houston *et al.*, 2018a; Kimmins *et al.*, 2024). Known risk factors include exposure to a variety of endocrine disrupting chemicals, pesticides, excessive heat, radiation, select medicines, a poor diet, and other gonadotoxic compounds, with numerous reports of their negative effects on sperm quality (Houston *et al.*, 2016, 2018b; Skakkebæk *et al.*, 2022; Kolaitis *et al.*, 2023; Kimmins *et al.*, 2024; Gillespie *et al.*, 2025). Such environmental and lifestyle factors are predicted to be key drivers of declining sperm counts that have been observed worldwide (Levine *et al.*, 2017, 2023; Kimmins *et al.*, 2024). While genetic and lifestyle factors are each considered to play key roles in the causality of male fertility, how such factors interact remains poorly understood and could have significant impact on infertility aetiology (Houston *et al.*, 2021a, b).

To better understand male infertility holistically, it is essential to understand the overlapping contributions of genetic and lifestyle factors. This includes investigating the consequence of genetic causes of male fertility that may cause subfertility, which could be compounded by lifestyle exposures, i.e. a two-hit scenario (Guma *et al.*, 2023; Chen *et al.*, 2025).

In recent years, there has been a notable emphasis on defining the genetic causes of male infertility (Oud *et al.*, 2020, 2021, 2022; Houston *et al.*, 2021c; Nagirnaja *et al.*, 2022). Meanwhile, a significant portion of the genetic causes of human male infertility, or subfertility, likely remain unidentified (Houston *et al.*, 2021c; Nagirnaja *et al.*, 2022). Compelling studies have revealed damaging bi-allelic genetic variants in *M1AP* as relatively frequently associated with human male infertility (Wyrwoll *et al.*, 2020; Rotte *et al.*, 2025). *M1AP* encodes meiosis 1 associated protein that promotes recruitment of TEX11 to the ZZS complex and aids in DNA recombination during prophase I of meiosis (De Muyt *et al.*, 2018; Li *et al.*, 2023; Rotte *et al.*, 2025). Of those men suffering from infertility with damaging *M1AP* variants that have been studied, 65% present with non-obstructive azoospermia due to meiotic arrest (Wyrwoll *et al.*, 2020; Rotte *et al.*, 2025). The remaining 35% of infertile men present with varying degrees of severe oligozoospermia (cryptozoospermia). This variability in genotype–phenotype relationship suggests that the effects of the loss of M1AP function during meiosis may be exacerbated by lifestyle factors, or equally, could be improved by the removal of negative environmental insults. This hypothesis is supported by the phenotype of *M1ap* knockout mice, which present with oligozoospermia due to meiotic defects and are subfertile (Arango *et al.*, 2013).

Of global relevance, obesity is prevalent in men of reproductive age (Welfare AIoHa, 2022; NIH, 2026) and is often caused by poor diet. As such, in this study, we aimed to explore the potential for an interaction between genetic and lifestyle factors on male infertility, using a subfertile *M1ap^−/−^* mouse model and an unhealthy Western-style high-fat and high-sugar diet. An additional cohort of mice was also aged on a standard (control) diet to explore the compounding effects of age and genotype on fertility. Our data reveal that a high-fat and high-sugar Western diet exacerbates subfertility in a *M1ap^−/−^* mouse model. Notably, for several parameters, a negative impact of Western diet was observed in the *M1ap^−/−^* mouse model but not in *M1ap^+/+^*, providing strong evidence for an interaction between genetic and lifestyle factors in male infertility aetiology that could be of relevance for human reproduction. Our proof-of-concept study highlights the complex factors affecting male fertility and supports a push towards a more holistic understanding of the causes of infertility, for improved treatment.

## Materials and methods

### Ethics statement

All experiments involving mice adhered to ethics guidelines published by the Australian National Health and Medical Research Council (NHMRC). Mouse experiments were approved by the University of Melbourne Animal Ethics Committee (applications 30244 and 32004). Mice were housed in standard conditions, at 22°C on a 14-h light cycle and 50–60% humidity, with standard environmental enrichment.

### M1AP expression analysis

Tissue expression data for *M1AP* and *M1ap* were collected from NCBI (Fagerberg *et al.*, 2014; Yue *et al.*, 2014).

### Knockout mouse production

Knockout mice were generated on a C57BL/6J background at the Monash Genome Modification Platform (Clayton, VIC, Australia). A sequence of 1517 bp, containing exon 4 and flanking intronic regions, was removed using CRISPR/Cas9 technology. To do so, the full length *M1ap* mouse transcript (ENSMUST00000113980.4) was targeted using CRISPR guide RNA sequences: ACGCAGTGTACATGCATGAG and CCGTCACATTTACGCTGCAT. Gene sequence changes were identified with Sanger sequencing. Mice heterozygous for the deletion were inter-crossed to produce homozygous individuals and wild type (*M1ap^+/+^*) controls. Genotyping was performed by Transnetyx (Corvoda, USA) using proprietary primers. *M1ap* transcript level was assessed via quantitative RT-PCR on testis cDNA relative to *Hprt* and *Ppia* (O’Bryan *et al.*, 2025). *M1ap* primers in the deleted region included 5′-GGAGCGTTCAGACTCACCAT-3′ forward and 5′-GATGATCCTTGGCTCTGGAA-3′ reverse. *M1ap* primers upstream of the deleted region included 5′-GTGAGGCCTTGCAGAACTTC-3′ forward and 5′-AGTTCCCTCTCACTTGCACA-3′ reverse. *Hprt* primers included 5′-AGTCCCAGCGTCGTGATTAG-3′ forward and 5′-TTTCCAAATCCTCGGCATAA-3′ reverse. *Ppia* primers included 5′-CGTCTCCTTCGAGCTGTTT-3′ forward and 5′-CCCTGGCACATGAATCCT-3′ reverse.

### Western diet and age treatment

To test the effects of poor diet on male fertility in wild type and *M1ap* mutant males, mice were fed *ad libitum* on a Western-style diet of 21% fat, 34.1% sugar, 19% protein (SF00-219, Specialty Feeds, Glen Forrest, WA, Australia), which has been shown to increase mouse body, liver, and subcutaneous fat weight compared to a standard, lower calorie diet of 7% fat, 10% sugar (Bodden *et al.*, 2022). In our study, control mice of both genotypes (*M1ap^+/+^* and *M1ap^−/−^*) were fed on BARASTOC mice breeder cube feed—8.5% fat, 25% starch, 20% protein (Ridley Corporation, Melbourne, VIC, Australia). Mice were fed on the specific diet from weaning until fertility assessment or dissection, as detailed below.

A separate cohort of mice were phenotyped at 26 weeks of age to separately investigate the combined effects of age and *M1ap* loss on male fertility. These mice were reared on the standard BARASTOC diet post-weaning.

### Fertility analysis

Mice were aged to 10 weeks of age (young cohort) or 26 weeks of age (aged cohort) and their fertility assessed using an established pipeline (Houston *et* *al.*, 2021a), with timing outlined in Fig. 1C. Briefly, at least 5 male mice of each genotype and treatment were set up to mate with two wild-type females each (6–12 weeks old). At least three males were used for aged breeding analyses. During the overnight mating period (until two females were successfully mated), males were co-housed with females and fed on the control diet, then placed back on their respective diets during the morning to afternoon period. Litter sizes were recorded as the number of pups generated per plug, where zero denotes a successful plug but no offspring. At 12 (young) or 28 (aged) weeks of age, males were culled and weighed. One testis and epididymis were dissected, weighed, and fixed in Bouin’s solution for 5 h then submerged in 70% ethanol until processing for histological assessment (Houston *et al.*, 2021a). Remaining testes and epididymides were snap frozen on dry ice for calculation of daily sperm production and epididymal sperm counts using the Triton-X-100 solubilization and sonication method as described previously (Dunleavy *et al.*, 2021). Gonadal fat pads were also weighed and fixed in Bouin’s solution for 5 h before histological processing as above, then stained with haematoxylin and eosin. Images were taken and adipocyte size (n ≥ 20 cells/animal) was measured using ImageJ (Bodden *et al.*, 2022). Liver and seminal vesicle weights were recorded.

**Figure 1.**
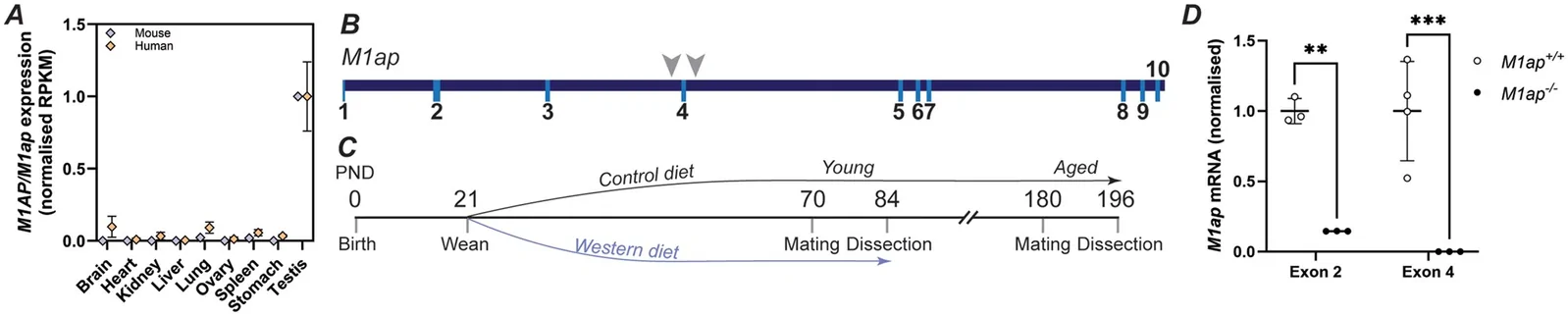
***M1ap* knockout design and experimental strategy.** (**A**) *M1AP/M1ap* expression in human and mouse tissues as highlighted by RNA sequencing. (**B**) Mouse *M1ap* gene map, noting exons 1–10. Grey arrows denote CRISPR cut sites for removal of exon 4. (**C**) Timeline for mouse experiments. Mice were weaned onto a control or Western diet and aged to 10 weeks before mating followed by dissection at 12 weeks of age. Mice on a control diet were also aged to ∼26 weeks before mating and dissection. (**D**) Testis *M1ap* expression was assessed in each genotype relative to *Hprt* and *Ppia* and then normalized to wild type (*M1ap*^+/+^) levels. White circles denote wild-type males (*M1ap^+/+^*) and black circles denote *M1ap* knockout males (*M1ap^−/−^*). **P < 0.01, ***P < 0.001.

For histological analyses, 5 µm tissue sections were cut. Testis sections were stained with periodic acid and Schiff’s reagents and haematoxylin. Epididymis sections were stained with haematoxylin and eosin. Round spermatid numbers were counted in Stage VIII seminiferous tubules (n ≥ 10 tubules/animal, n ≥ 3/genotype) (Houston *et al.*, 2022b). Germ cell apoptosis was assessed by dual staining testis sections with cleaved-Caspase 3 and cleaved-Caspase 7 as detailed previously (Dunleavy *et al.*, 2023). The number of positive cells was counted per seminiferous tubule (n = 50/animal, n = 3–4/genotype). Data are presented as the average number of positive cells per tubule per biological replicate (per mouse), but each data point per mouse was used for statistical analysis using a zero-inflated negative binomial regression (zinb package) in RStudio (2025.09.2 Build 418, USA). The number of positive cells was fit into the model as a response variable. Genotype and diet and their interaction were assigned as fixed effects and animal identifier as a random effect.

### Sperm motility and morphology analysis

Sperm were collected from the cauda epididymis via retrograde air perfusion of the vas deferens using the backflushing technique (Houston *et al.*, 2021b). Sperm were resuspended in MT6 medium at 37°C for 10 min prior to motility assessment via computer-assisted semen analysis (Houston *et al.*, 2021b). Remaining sperm were washed and diluted in 1 × PBS then dried onto SuperFrost slides (Epredia, USA). Sperm were fixed in 90% ethanol for 2 min, washed in water and stained with Mayer’s haematoxylin for 10 min, Scott’s tap water substitute for 2 min and eosin (Amber Scientific, Australia) for 1 min to investigate sperm structure. Sperm mid-piece and full tail length measurements were measured using ImageJ (version 1.52k, USA). Sperm head shape normality was assessed manually as a binary normal or abnormal (Houston *et al.*, 2024a), noting no abnormalities in sperm tail development were observed.

### Statistical analysis

Statistical analyses were performed in Prism 10 (GraphPad, San Diego, USA) or RStudio (described above). A two-way ANOVA was undertaken to determine the effects of genotype, diet or an interaction between both factors, on each biological parameter. A *post hoc* Dunnett’s multiple comparison was used to compare genotypes on the same diet or between the same genotype on a different diet. When comparing between two genotypes alone, data were assessed via an unpaired Student’s *t*-test. A *P*-value <0.05 was considered statistically significant.

## Results

### Establishment of experimental models

In this study, we aimed to explore the combined impacts of a genetic and a lifestyle factor on male fertility using a subfertile *M1ap^−/−^* mouse model. *M1AP* expression is highly testis-enriched in adult humans and mice (Fig. 1A) and M1AP has an established role in male meiosis (Arango *et al.*, 2013). To generate our loss of function *M1ap* model, we used CRISPR to remove exon 4 of the full length *M1ap* coding transcript, introducing a premature stop codon in exon 5 (Fig. 1B). The *M1ap* deletion was confirmed by Sanger sequencing and by quantitative RT-PCR using primers targeting exon 4 (Fig. 1D, *P *< 0.001). Primers targeting exon 2 (upstream of the deletion) revealed *M1ap* expression was reduced by 86% in the knockout model (Fig. 1D, *P *< 0.01), where any residual transcripts would have little to no protein function. These data reveal our *M1ap* mouse model recapitulates the biological situation of men containing damaging *M1AP* genetic variants. *M1ap^+/+^* (wild type) and *M1ap^−/−^* (knockout) mice were then fed on a control or Western diet prior to mating at 10 weeks of age, followed by dissection at 12 weeks of age (Fig. 1C).

We first sought to assess the effect of Western diet treatment on body weight and fat mass in male *M1ap^+/+^* mice (Supplementary Fig. S1). Exposure to a Western diet led to a significant increase in body weight (33.12 in Western versus 28.9 g in control, *P *= 0.0002), gonadal fat pad weight (566.2 versus 249.7 mg, *P *< 0.0001) and adipocyte size (5135 versus 2982 µm^2^, *P *< 0.0001), and liver weight (1.85 versus 1.41 g, *P *= 0.0027). These data replicate a previous study (Bodden *et al.*, 2022).

### Male fertility is impacted by both the loss of M1AP and a Western diet

The loss of *M1ap* in male mice on a control diet resulted in subfertility (Fig. 2A, Supplementary Fig. S2A), highlighted by a 70% reduction in litter size (2.5 pups/litter) compared to *M1ap^+/+^* males (8.25 pups/litter, *P *< 0.0001). Notably, the loss of *M1ap* also resulted in a 50% reduction in pregnancy rate per copulatory plug (Fig. 2B). Equally, male fertility was negatively impacted in *M1ap^+/+^* mice fed a Western diet (Fig. 2A). Specifically, *M1ap^+/+^* males on a Western diet produced litters sizes on average 37% smaller than those on a control diet (5.2 pups/litter in Western diet versus 8.25 in control, *P *= 0.015) but no effects on pregnancy rate were observed (Fig. 2B). Notably, the combined effect of *M1ap* loss and a Western diet further exacerbated subfertility in *M1ap^−/−^* males (Fig. 2A). While *M1ap^−/−^* males on a control diet produced 2.5 pups/litter, those exposed to a Western diet produced an average litter size of 0.37 pups/litter (*P *= 0.015). This equates to a 98% reduction in litter size compared to *M1ap^+/+^* males raised on a control diet. Similarly, when *M1ap^−/−^* males were fed on a Western diet, pregnancy rate was reduced by 82% compared to *M1ap^+/+^* males on a control diet. These data support a negative interaction between *M1ap* loss and diet on male fertility.

**Figure 2.**
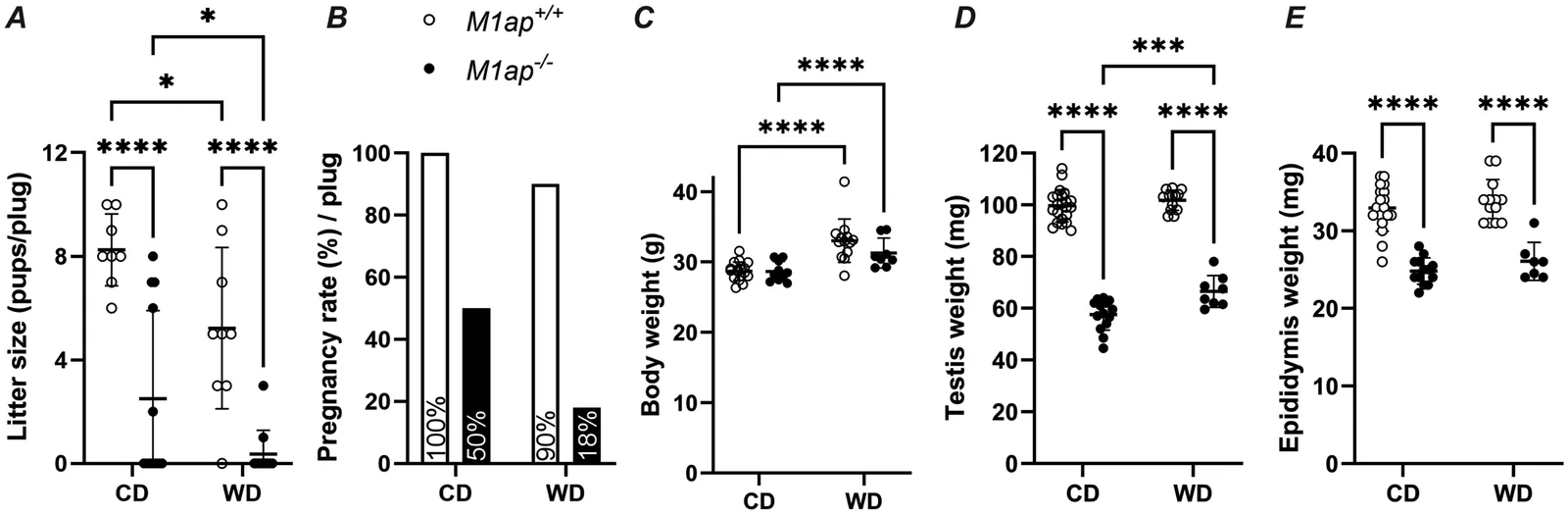
**M1AP and diet modulate optimal male fertility in mice.** (**A**) Litter size measured following plug mating, where 0 denotes no pregnancy. (**B**) Pregnancy rate as a percentage of all successful mating attempts where the female became pregnant. (**C**) Body weight. (**D**) Testis weight. (**E**) Epididymis weight. White circles denote wild type males (*M1ap^+/+^*) and black circles denote *M1ap* knockout males (*M1ap^−/−^*). Abbreviations: CD = control diet; WD = Western diet. ****P < 0.0001, ***P < 0.001, *P < 0.05.

At a whole animal level, the deletion of *M1ap* did not impact body weight compared to *M1ap^+/+^* males (Fig. 2C, *P *= 0.95). The Western diet increased body weight in *M1ap^+/+^* males by 15% compared to control animals (*P *< 0.0001). A similar increase in body weight was measured in *M1ap^−/−^* males on a Western diet compared to those on a control diet (*P *= 0.01), highlighting that diet but not the presence of M1AP modulates body weight. As reported previously (Arango *et al.*, 2013), the absence of *M1ap* resulted in a substantial reduction in testis weight compared to *M1ap^+/+^* males (Fig. 2D, *P *< 0.0001). Aligning with this reduction in testis weight, epididymis weight was significantly reduced in *M1ap^−/−^* males on a control diet (Fig. 2E, *P *< 0.0001). Testis and epididymis weights were not significantly changed by exposure to a Western diet in *M1ap^+/+^* males (Fig. 2D and E). While testis weights of *M1ap^−/−^* males fed a Western diet were significantly reduced compared to *M1ap^+/+^* males of both diet groups (*P *< 0.0001), they were 15% heavier than *M1ap^−/−^* males fed a control diet (Fig. 2D, *P *= 0.0008).

A separate cohort of *M1ap^+/+^* and *M1ap^−/−^* males was aged to 26 weeks on a control diet before mating and dissection (Supplementary Fig. S2). Consistent with previous studies (Hu *et al.*, 2018; Houston *et al.*, 2022a,b; O’Bryan *et al.*, 2025), ageing mice did not significantly impact the fertility of *M1ap^+/+^* males. Litter sizes (*P *= 0.12, Supplementary Fig. S2B and C), and testis (*P *= 0.99, Supplementary Fig. S2D) weight were comparable to young *M1ap^+/+^* males, while epididymis weight was moderately increased (*P *= 0.049, Supplementary Fig. S2E). Examination of aged *M1ap* males, revealed that age did not exacerbate the *M1ap^-/-^* phenotype. *M1ap^-/-^* males were subfertile and produced litter sizes comparable to young *M1ap^-/-^* males (4.8 pups/litter in aged *M1ap^−/−^* versus 2.5 in young, Supplementary Fig. S2C), *P *= 0.17 (comparison not graphed). As was seen in younger animals, the testis weights of aged *M1ap^−/−^* males was significantly reduced compared to aged-matched controls. Weights were comparable to those of young *M1ap^−/−^* males (*P *= 0.77, Supplementary Fig. S2B). Epididymis weights in aged *M1ap^−/−^* males were similarly reduced compared to aged *M1ap^+/+^* controls. Like as was observed for aged *M1ap^+/+^* males, epididymis weight in *M1ap^−/−^* males increased compared to young *M1ap^−/−^* males (*P *= 0.01, Supplementary Fig. S2C). Collectively, these results highlighted that diet but not age plays a significant role in the severity of infertility in *M1ap^−/−^* males.

### M1AP is required for meiosis and quantitatively normal sperm production

We next investigated spermatogenesis in young *M1ap^+/+^* and *M1ap^−/−^* males fed a Western and control diet (Fig. 3, Supplementary Fig. S3A and B). In *M1ap^+/+^* mice fed on a control diet, sperm production was complete and robust (Fig. 3A, Supplementary Fig. S3A). By contrast, a severe hypospermatogenesis phenotype (H) was observed in males lacking *M1ap* (Fig. 3A), characterized by defects in spermatocytes undergoing meiosis (arrows), consistent with previous findings (Arango *et al.*, 2013). Defects in *M1ap^−/−^* control diet males included germ cell death (Supplementary Fig. S3B and E, notably in developmentally arrested metaphase spermatocytes), sperm head shaping abnormalities (Supplementary Fig. S3F), vacuoles in the seminiferous epithelium (Supplementary Fig. S3G), and germ cell sloughing (Supplementary Fig. S3H). While spermatogenesis was largely normal in *M1ap^+/+^* males fed a Western diet, there was an increased frequency of pyknotic (dying) germ cells that were frequently present near the lumen of tubules (Fig. 3A inset, arrows, Supplementary Fig. S3C). *M1ap^−/−^* males fed a Western diet presented with similar defects to those on a control diet. Meiosis abnormalities (arrows) including germ cell sloughing and epithelial vacuolation, leading to hypospermatogenesis, were notably observed (Supplementary Fig. S3D). We quantified the frequency of these defects across tubules in each treatment group (Fig. 3B–D). Germ cells with abnormal morphology (abnormal chromatin structure) were observed 210% more frequently in *M1ap^−/−^* males compared to *M1ap^+/+^* on a control diet (Fig. 3B, *P *< 0.0001). Abnormal germ cells were also detected in *M1ap^+/+^* males fed on a Western diet, which were 183% more common than in *M1ap^+/+^* males on a control diet (*P *< 0.0001). Feeding *M1ap^−/−^* males a Western diet exacerbated the frequency of abnormal germ cells by 25% compared to control diet-fed *M1ap^−/−^* males (Fig. 3B, *P *= 0.0031) and 37% compared to *M1ap^+/+^* males on a Western diet. A similar finding was observed for germ cell sloughing (Fig. 3C), which was elevated by 189% in *M1ap^−/−^* males compared to *M1ap^+/+^* (*P *= 0.017) on a control diet. A Western diet increased germ cell sloughing in *M1ap^+/+^* males, which was elevated by 181% compared those on a control diet (*P *= 0.021). Sloughing was also exacerbated in the combination group, where feeding *M1ap^−/−^* males a Western diet increased sloughing by 127% compared to *M1ap^−/−^* males on a control diet (*P *= 0.0003) and 133% compared to *M1ap^+/+^* males on a Western diet (*P *= 0.0002). The presence of vacuoles in the seminiferous epithelium was elevated in *M1ap^−/−^* males fed on a control diet (*P *= 0.034, Fig. 3D) but not *M1ap^+/+^* males fed a Western diet. Feeding *M1ap^−/−^* males a Western diet further elevated the frequency of vacuoles (*P *= 0.0008, Fig. 3D) compared to *M1ap^−/−^* on a control diet, thus worsening the hypospermatogenesis phenotype. These findings were further evidenced by a 554% elevation in apoptotic germ cells in *M1ap^−/−^* males fed a control diet, compared to *M1ap^+/+^* males (Fig. 3E, *P *< 0.0001). While feeding *M1ap^+/+^* males a Western diet increased germ cell apoptosis by 98% (*P *= 0.011), the diet challenge did not exacerbate germ cell apoptosis in *M1ap^−/−^* males fed a Western diet compared to *M1ap^−/−^* males on the control diet (*P *= 0.99). These findings highlight that exposure to a Western diet will exacerbate the presence of abnormal germ cells, sloughing and vacuoles in the presence of *M1ap^−/−^* loss-of-function.

**Figure 3.**
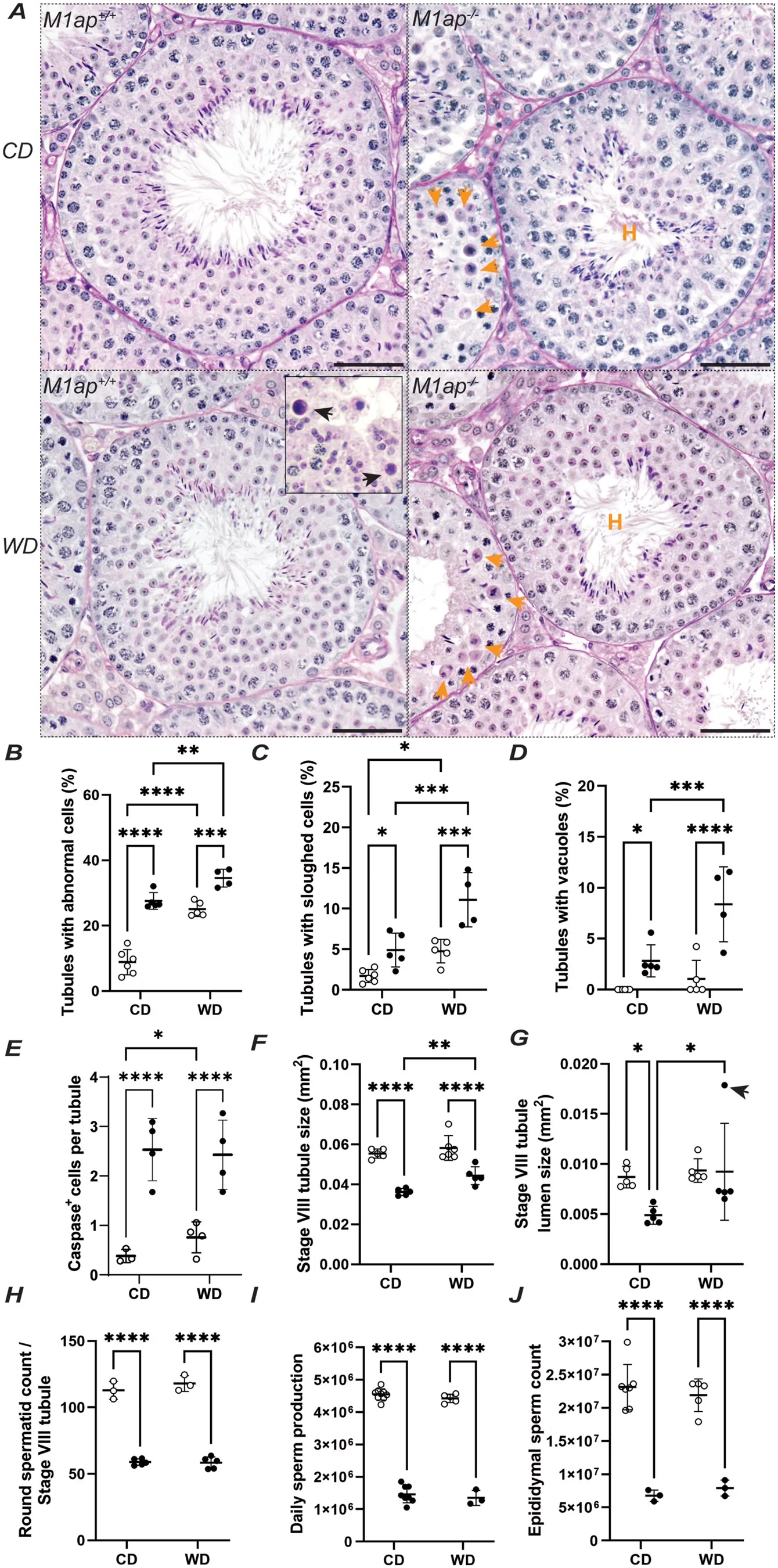
**A Western diet exacerbates the presence of abnormal germ cells in the testis of *M1ap^−/−^* males.** (**A**) Testis histology. Orange arrows indicate dying spermatocytes, orange Hs indicate hypospermatogenic tubules. Inset: black arrows indicate abnormal dying cells. Scale bars = 50 µm. (**B–D**) The percentage of tubules in which abnormal cells, sloughed cells, or vacuoles, respectively. (**E**) Caspase positive cells per tubule. (**F–H**) Stage VIII tubule size, lumen size, and round spermatid count, respectively. (**I**) Daily sperm production. (**J**) Epididymal sperm count. White circles denote wild-type males (*M1ap^+/+^*) and black circles denote *M1ap* knockout males (*M1ap^−/−^*). Abbreviations: CD = control diet; WD = Western diet. ****P < 0.0001, ***P < 0.001, **P < 0.01, *P < 0.05.

In agreement with the germ cell loss in young *M1ap^−/−^* testes, testis tubule area was reduced by 35% compared to *M1ap^+/+^* males in the control diet treatment group (Fig. 3F, *P *< 0.001). The Western diet did not affect tubule area size in *M1ap^+/+^* males (*P *= 0.70). Notably, tubule size in *M1ap^−/−^* males fed a Western diet was 23% smaller than *M1ap^+/+^* males (*P *< 0.0001), but 22% larger than *M1ap^−/−^* males on a control diet (*P *= 0.003). This increase in tubule area in the combination group compared to *M1ap^−/−^* control diet males is consistent with the elevated testis weight data above (Fig. 2D). The cause for this elevation was found to be an 89% increase in lumen size in *M1ap^−/−^* males fed a Western versus control diet (*P *= 0.017, Fig. 3G), suggestive of fluid retention due to abnormal efferent duct function (Eddy *et al.*, 1996; Oh *et al.*, 2023). While all *M1ap^−/−^* western diet mice displayed elevated lumen size compared to *M1ap^−/−^* males on a control diet, one male had seminiferous tubule lumen area that was twice the area of *M1ap^+/+^* males (Fig. 3G—arrow, Supplementary Fig. S4). Compared to *M1ap^+/+^* males, very few sperm were apparent within the cauda epididymis of this male (Supplementary Fig. S4E and F). Deletion of *M1ap* significantly reduced round spermatid numbers compared to *M1ap^+/+^* males in both diet groups (*P *< 0.0001, Fig. 3H). Numbers were unchanged in *M1ap^+/+^* fed a Western diet compared to control diet, and the Western diet did not worsen the reduction in round spermatids in the *M1ap^−/−^* model compared to the control diet. This pattern was mirrored in daily sperm production (Fig. 3I) and epididymal sperm counts (Fig. 3J), which were significantly reduced in *M1ap^−/−^* males fed on a control diet compared to *M1ap^+/+^* males on a control diet (*P *< 0.0001) but not affected in *M1ap^+/+^* males on a Western diet. Exposure to a Western diet did not further exacerbate the reductions in daily sperm production (*P *= 0.61) or epididymal sperm count (*P *= 0.42) observed in *M1ap^−/−^* males.

In *M1ap^+/+^* males on a control diet, the epididymis was replete with sperm and sloughed germ cells were not detected (Fig. 4A). Consistent with findings in the testis, fewer sperm were present in the epididymis of *M1ap^−/−^* males on a control diet and immature sloughed germ cells were frequently observed, at a higher rate than in *M1ap^+/+^* males on a control diet (Fig. 4B; *P *< 0.0001). No noticeable effects of a Western diet were observed on epididymis histology in *M1ap^+/+^* males (Fig. 4A). However, the Western diet exacerbated the frequency of abnormal immature sloughed germ cells in the epididymides of *M1ap^−/−^* males (Fig. 4B; *P *= 0.0027).

**Figure 4.**
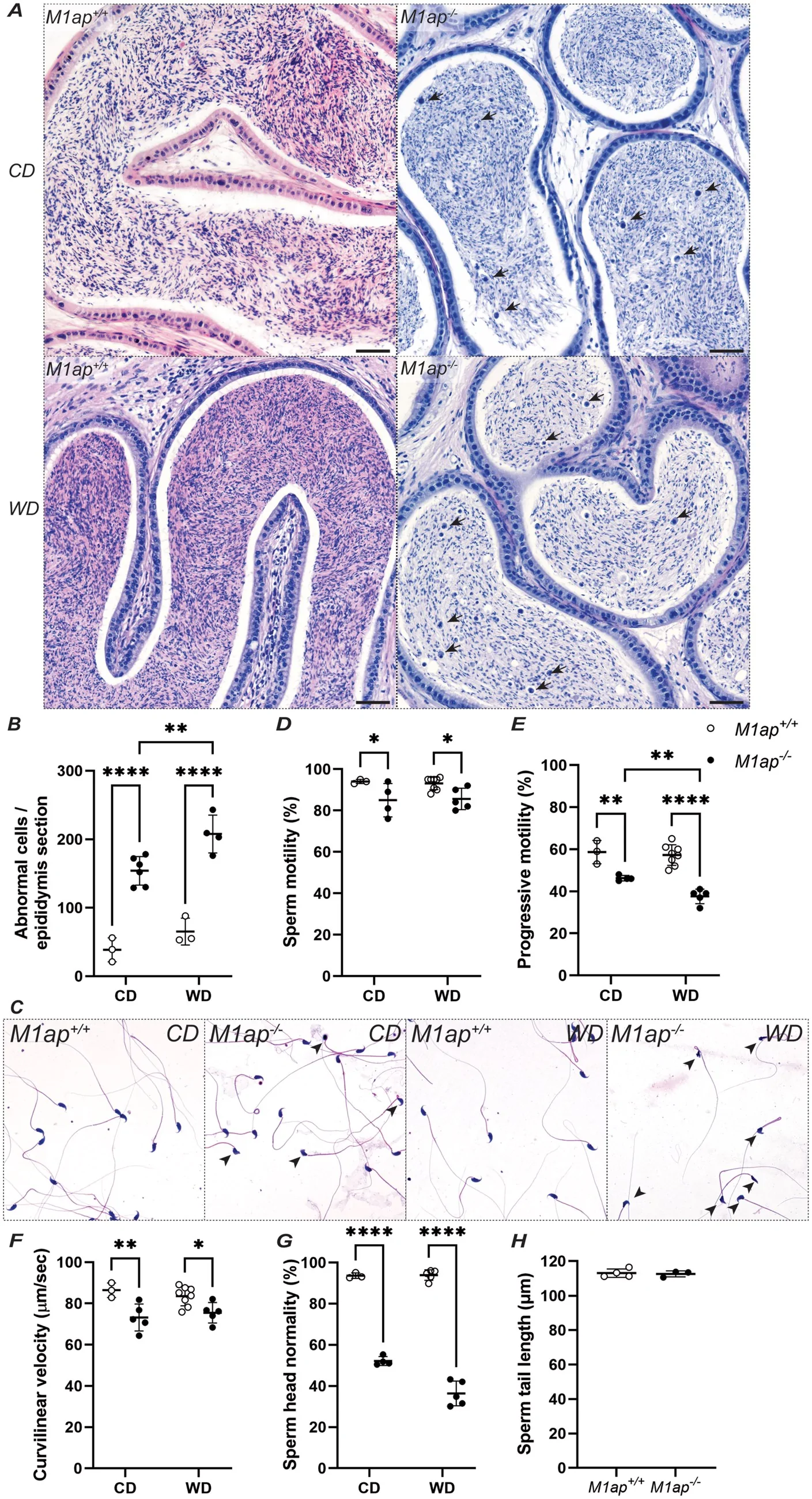
**A Western diet worsens deficits in progressive motility and sperm head morphology observed in *M1ap^−/−^* males.** (**A**) Cauda epididymis histology. Arrows indicate premature and abnormal germ cells in *M1ap^−/−^* males. Scale bars = 50 µm. (**B**) Quantification of immature germ cells in the epididymis. (**C**) Sperm morphology. (**D**) Sperm motility. (**E**) Sperm progressive motility. (**F**) Sperm curvilinear velocity; each measured by computer-assisted semen analysis. (**G**) The percentage of sperm with normal head shape. (**H**) Sperm tail length. White circles denote wild-type males (*M1ap^+/+^*) and black circles denote *M1ap* knockout males (*M1ap^−/−^*). Abbreviations: CD = control diet; WD = Western diet. ****P < 0.0001, **P < 0.01, *P < 0.05.

We next explored the effects of a gene and diet interaction on sperm quality. This analysis revealed striking abnormalities in sperm head shape in sperm from *M1ap^−/−^* males compared to *M1ap^+/+^* males on a control diet (Fig. 4C and G). Specifically, 52% of sperm from *M1ap^−/−^* males were normally shaped, compared to 94% in *M1ap^+/+^* on a control diet (*P *< 0.0001). In *M1ap^+/+^* males, the incidence of sperm morphological abnormalities was unaffected by exposure to a Western diet (*P *= 0.92). By contrast, the negative impact of *M1ap* loss on sperm morphology was significantly exacerbated by a Western diet (Fig. 4C and G), where only 36% of sperm were normally shaped (*P *< 0.0001 compared to all other groups). Further, the loss of *M1ap^−/−^* significantly reduced total sperm motility compared to *M1ap^+/+^* males on a control diet (Fig. 4D, *P *= 0.026). No effects of a Western diet on total sperm motility were, however, seen in *M1ap^+/+^* males (*P *= 0.79), nor did the Western diet worsen motility in *M1ap^−/−^* males (*P *= 0.85) compared to *M1ap^−/−^* males on the control diet. By contrast, progressive motility (Fig. 4E) was significantly reduced in *M1ap^−/−^* males on a control diet compared to *M1ap^+/+^* males (46% versus 59%, *P *= 0.001). While the Western diet had no effect on progressive motility in *M1ap^+/+^* males (*P *= 0.62), it worsened progressive motility in *M1ap^−/−^* males compared to those fed a control diet (38% versus 46%, *P *= 0.007). Finally, the loss of *M1ap* impaired curvilinear velocity (*V*_CL_) by 15% compared to *M1ap^+/+^* males (Fig. 4F, *P *= 0.003). No effects of the Western diet treatment on *V*_CL_ were observed in *M1ap^+/+^* (*P *= 0.42) or *M1ap^−/−^* males (*P *= 0.49). Sperm tail length was unaffected by genotype and diet treatment (Fig. 4H). Together, these data evidence the existence of genotype and environment interactions influencing key sperm parameters required for male fertility.

## Discussion

In this study, we aimed to test the individual and combined effects of a genetic and lifestyle factor in the severity of male infertility, as a proof-of-concept for genetic and lifestyle/environmental interactions more broadly. We used a *M1ap* loss of function mouse model, as damaging variants in *M1AP* have been relatively commonly associated with human male infertility (Wyrwoll *et al.*, 2020; Khan *et al.*, 2025; Rotte *et al.*, 2025). The genetic manipulation of *M1ap* in our mouse reflects the damaging effects of loss-of-function variants discovered in these men. There is also evidence of a spectrum of severity in infertility presentation in such men, suggestive of an additional component to their infertility aetiology. While in wild type (control) male mice a Western diet alone did not significantly compromise sperm parameters, including total motility, morphology and count in this study, it did compromise male fertility compared to controls as evidenced by a reduced litter size. Exposure to a Western diet exacerbated several genetically induced abnormalities in the *M1ap^−/−^* model, including the incidence of germ cell death and abnormal germ cell morphology, immature germ cells within the epididymis and sperm progressive motility. Collectively, these data demonstrate that genetic factors and environmental exposures can interact to negatively impact male fertility. Specifically, in the absence of *M1ap* function, exposure to a Western diet pushes sperm number and function significantly further below the fertility threshold, specifically 0.37 pups/litter compared to 2.5 on a control diet. The revelation of this interaction provides weight to exploring dietary interventions for men carrying damaging *M1AP* variants who present for fertility assessment, prior to more invasive medically assisted reproduction interventions.

M1AP functions during meiosis and contributes to DNA recombination by stabilizing ZZS complex intermediates (Li *et al.*, 2023). In its absence, as shown here and previously (Arango *et al.*, 2013), a significant portion of spermatocytes arrest at metaphase. While substantial germ cell arrest occurs in humans and mice lacking M1AP (Arango *et al.*, 2013; Wyrwoll *et al.*, 2020; Rotte *et al.*, 2025), it is not absolutely essential for male fertility, as indicated by subfertility in knockout mice and a spectrum of clinical presentations in infertile men (Arango *et al.*, 2013; Wyrwoll *et al.*, 2020; Khan *et al.*, 2025; Rotte *et al.*, 2025). There also may be fertile men with variants in *M1AP* who have not been considered due to selection bias but are potentially susceptible to certain environmental factors, which could be explored in future studies. Further, an unhealthy lifestyle may also worsen spermatogenesis in men with bi-allelic loss-of-function *M1AP* variants, leading to non-obstructive azoospermia. Collectively, while our study adds strong evidence for gene–lifestyle interactions contributing to male infertility aetiology, equivalent studies must be replicated in humans before any clinically applicable conclusions can be made. This includes directly testing whether dietary interventions on infertile men robustly alters semen profiles.

Globally men of reproductive age are being constantly exposed to numerous lifestyle factors that are known to impact spermatogenesis and male fertility (Kimmins *et al.*, 2024). The additive effects of these factors in disrupting molecular pathways, shared by men and animals, should be systematically tested so as to inform clinical interventions. Doing so will improve our understanding of the aetiology of infertility. While not tested in this study, it is anticipated that reverting to a healthy diet would improve fertility in mice and men lacking M1AP. We note that a cycle of spermatogenesis and epididymal maturation in man takes approximately 3 months (WHO, 2025). Fortunately, lifestyle factors such as diet can easily be modified, therefore removing the environmental challenge may shift men (and mice) men with certain genetic susceptibility to infertility or subfertility back into the fertile range, thus avoiding the need for medically assisted reproduction.

## Supplementary Material

deag130_Supplementary_Figure_S1

deag130_Supplementary_Figure_S2

deag130_Supplementary_Figure_S3

deag130_Supplementary_Figure_S4

## Data Availability

The data underlying this article will be shared on reasonable request to the corresponding author.
