# Supplementary material for: A Western-style diet exacerbates male infertility in M1ap knockout mice
Source: Hum Reprod. 2026 Aug 31;41(10):1633–42. doi: 10.1093/humrep/deag130 (PMC13635463; doi:10.1093/humrep/deag130)
Supplement: Supplementary file 1 [file deag130_supplementary_figure_s1.pdf]

Human Reproduction, 2026, 1–1
https://doi.org/10.1093/humrep/deag130
Supplementary Data

human reproduction
OXFORD

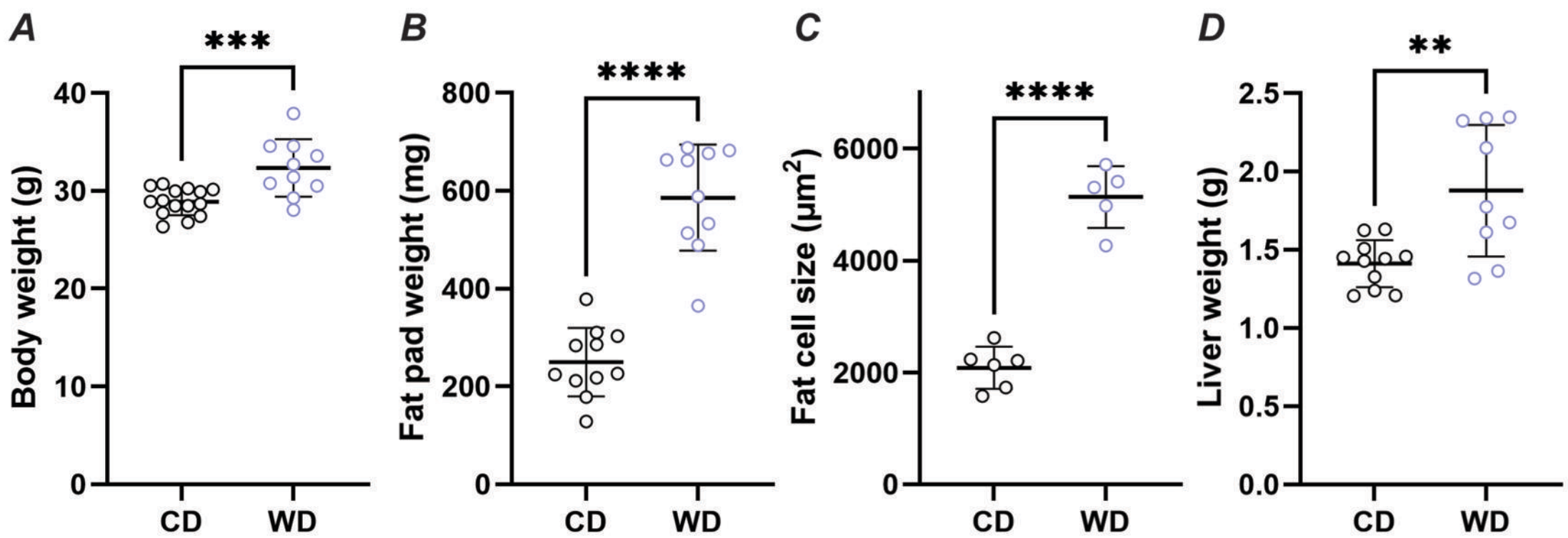

Supplementary Figure S1. Western diet validation. (**A**) Body weight. (**B**) Epididymal fat pad weight. (**C**) Fat cell size. (**D**) Liver weight. White circles indicate wild type mice on a control diet and blue circles indicate wild type mice on the Western diet. ****$P < 0.0001$, ***$P < 0.001$, **$P < 0.01$.
