# Supplementary material for: A Western-style diet exacerbates male infertility in M1ap knockout mice
Source: Hum Reprod. 2026 Aug 31;41(10):1633–42. doi: 10.1093/humrep/deag130 (PMC13635463; doi:10.1093/humrep/deag130)
Supplement: Supplementary file 2 [file deag130_supplementary_figure_s2.pdf]

Human Reproduction, 2026, 1–1
https://doi.org/10.1093/humrep/deag130
Supplementary Data

human reproduction
OXFORD

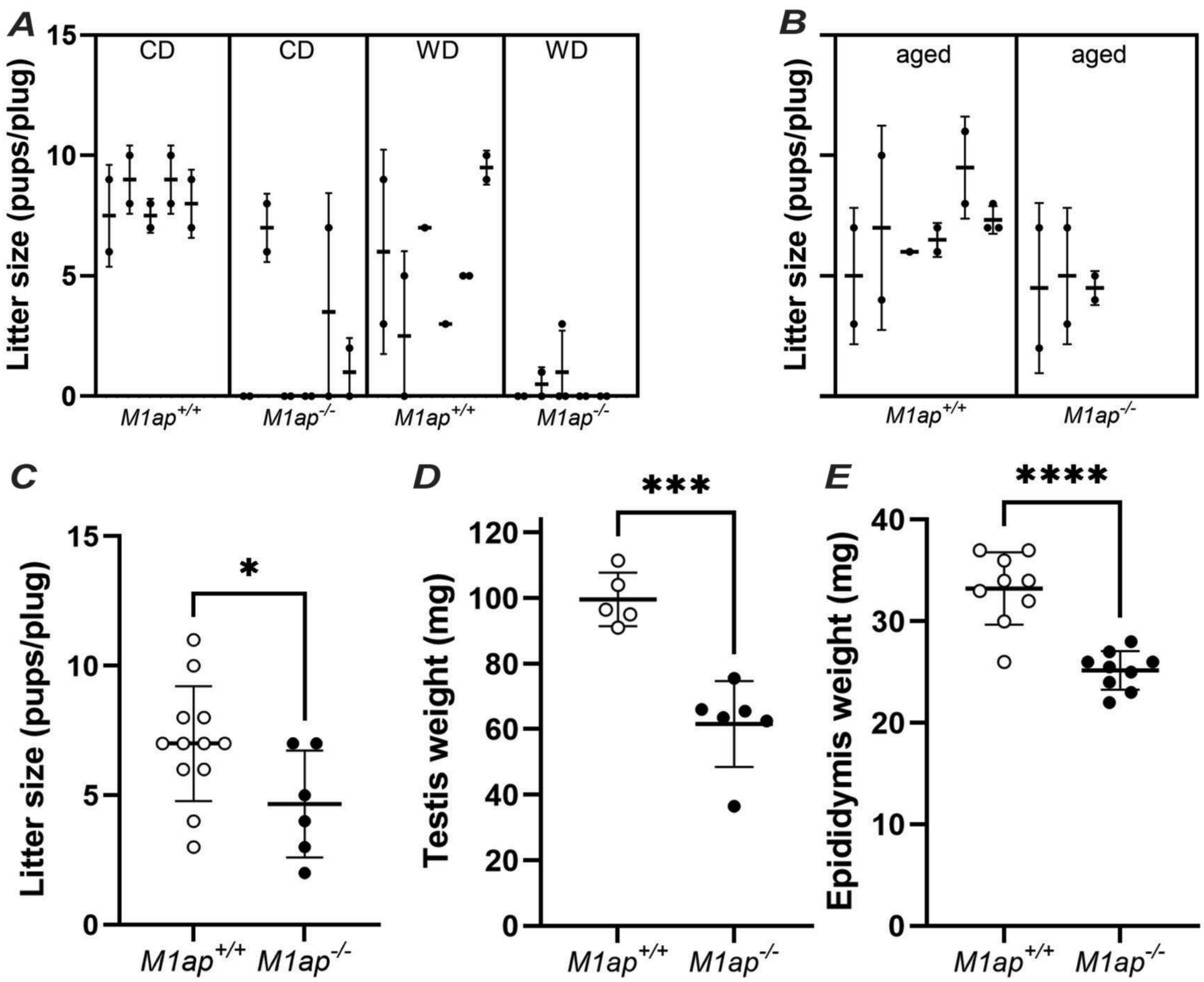

Supplementary Figure S2. Ageing does not worsen subfertility in *M1ap*$^{-/-}$ mice. Litter sizes for (**A**) young and (**B**) aged males in each treatment group were plotted per male to show variation in *M1ap*$^{-/-}$ males, where 0 denotes no pregnancy. CD = control diet, WD = Western diet. Aged male data are also shown for: (**C**) litter size measured following plug mating, (**D**) testis weight and (**E**) epididymis weight. White circles denote wild type males (*M1ap*$^{+/+}$) and black circles denote *M1ap* knockout males (*M1ap*$^{-/-}$). Except for in (A), mice were assessed at 6 months of age. ****$P < 0.0001$, ***$P < 0.001$, *$P < 0.05$.
