# Supplementary material for: A Western-style diet exacerbates male infertility in M1ap knockout mice
Source: Hum Reprod. 2026 Aug 31;41(10):1633–42. doi: 10.1093/humrep/deag130 (PMC13635463; doi:10.1093/humrep/deag130)
Supplement: Supplementary file 3 [file deag130_supplementary_figure_s3.pdf]

Human Reproduction, 2026, 1–1
https://doi.org/10.1093/humrep/deag130
Supplementary Data

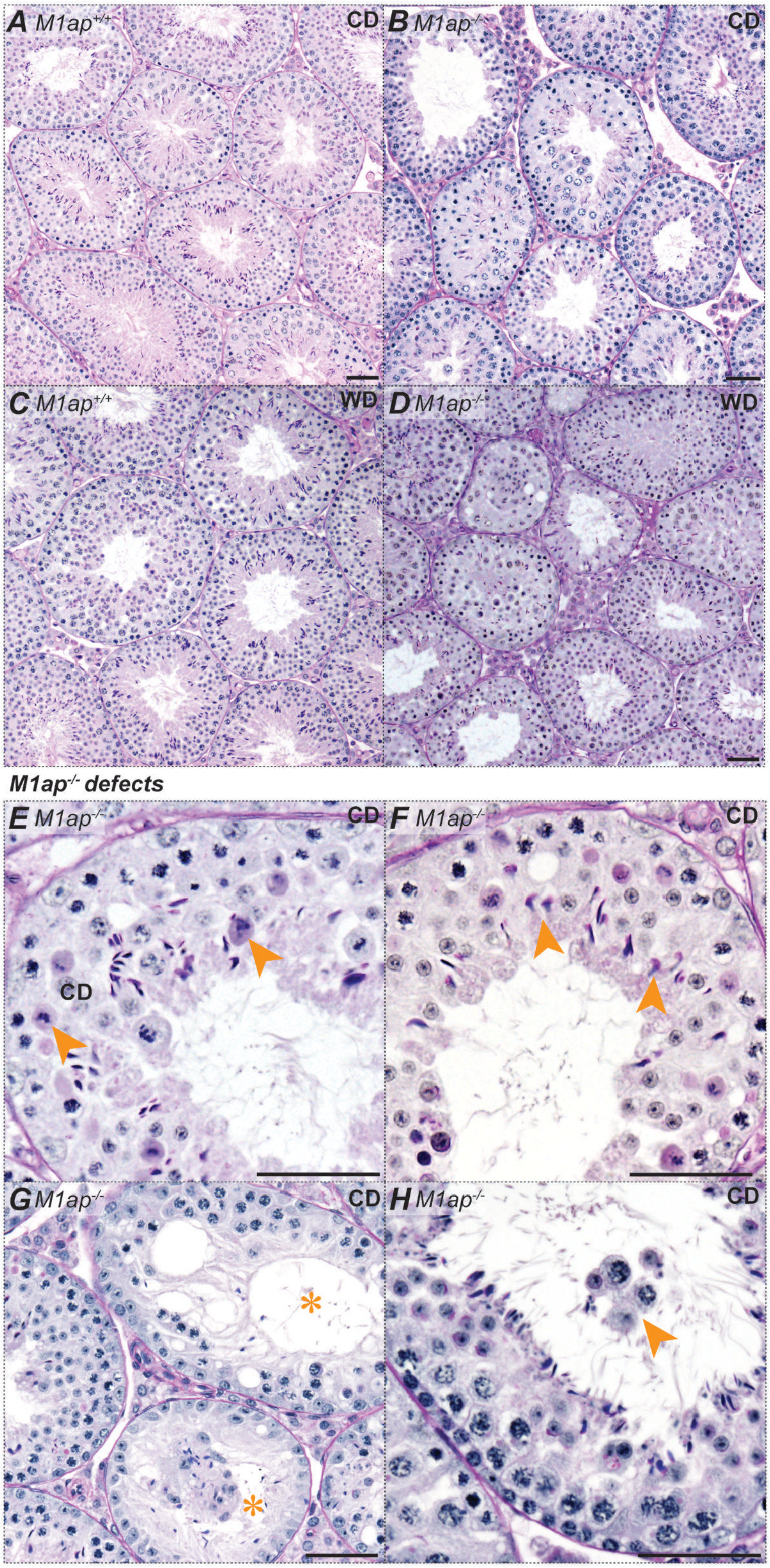

Supplementary Figure S3. M1AP is required for normal male meiosis, leading to severe spermatogenesis defects *M1ap*$^{-/-}$ mice. Testis sections from *M1ap*$^{+/+}$ (**A**) and *M1ap*$^{-/-}$ (**B**) on a control diet and *M1ap*$^{+/+}$ (**C**) and *M1ap*$^{-/-}$ (**D**) on a Western diet. CD = control diet, WD = Western diet. Abnormalities in *M1ap*$^{-/-}$ are further shown in (**E**) defects in chromosome segregation, (**F**) sperm head shape defects, (**G**) vacuoles (*) in the seminiferous tubules and (**H**) germ cell sloughing. Scale bars = 50 μm.
