# Supplementary material for: A Western-style diet exacerbates male infertility in M1ap knockout mice
Source: Hum Reprod. 2026 Aug 31;41(10):1633–42. doi: 10.1093/humrep/deag130 (PMC13635463; doi:10.1093/humrep/deag130)
Supplement: Supplementary file 4 [file deag130_supplementary_figure_s4.pdf]

Human Reproduction, 2026, 1–1
https://doi.org/10.1093/humrep/deag130
Supplementary Data

human reproduction OXFORD

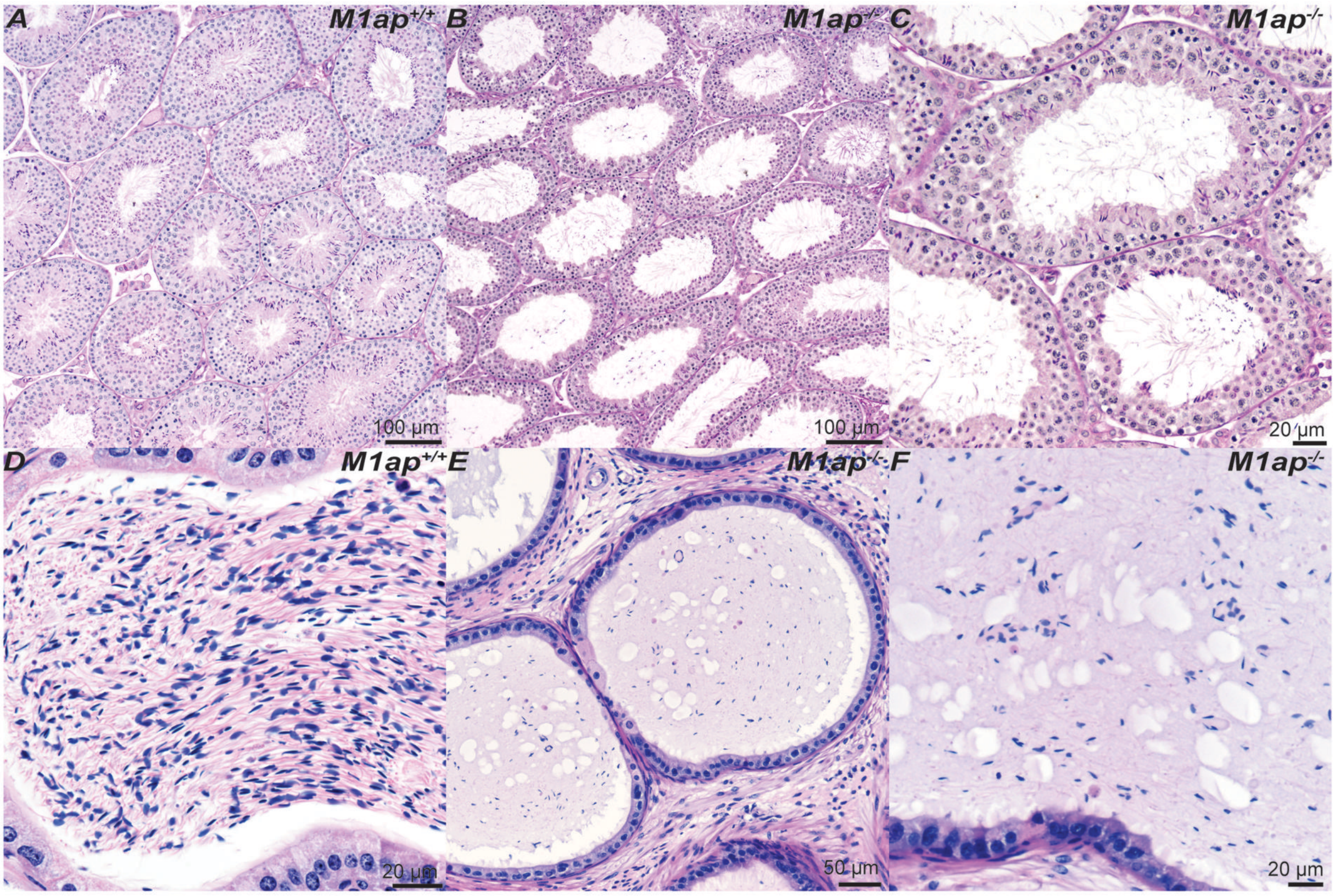

Supplementary Figure S4. Defects in reproductive tract fluid regulation identified in a *M1ap*$^{-/-}$ male fed a Western diet. (**A, D**) *M1ap*$^{+/+}$ (control) testis and epididymis histology. (**B, C**) *M1ap*$^{-/-}$ testis and (**E, F**) *M1ap*$^{-/-}$ epididymis histology, depicting enlarged lumen size and reduced sperm concentration.

© The Author(s) 2026. Published by Oxford University Press on behalf of European Society of Human Reproduction and Embryology.
This is an Open Access article distributed under the terms of the Creative Commons Attribution License (https://creativecommons.org/licenses/by/4.0/), which permits unrestricted reuse, distribution, and reproduction in any medium, provided the original work is properly cited.
Human Reproduction, 2026, 00, 1–14
https://doi.org/10.1093/humrep/deag130
Original Article
